# RECAPP-XPR: A smartphone application for presenting and recalling experimentally controlled stimuli over longer timescales

**DOI:** 10.3758/s13428-018-1157-x

**Published:** 2018-12-10

**Authors:** Cathleen Cortis Mack, Michael Harding, Nigel Davies, Geoff Ward

**Affiliations:** 1 Department of Psychology, University of Essex, Wivenhoe Park, Colchester, Essex UK; 2 Department of Computing and Communications, University of Lancaster, Bailrigg, Lancashire UK

**Keywords:** Smartphone, Free recall, Serial position curves, Temporal contiguity effects

## Abstract

We report two experiments that used smartphone applications for presenting and recalling verbal stimuli over extended timescales. In Experiment 1, we used an iPhone application that we had developed, called RECAPP-XPR, to present 76 participants with a single list of eight words presented at a rate of one word every hour, followed by a test of free recall an hour later. The experiment was exceptionally easy to schedule, taking only between 5 and 10 min to set up using a web-based interface. RECAPP-XPR randomly samples the stimuli, presents the stimuli, and collects the free recall data. The stimuli disappear shortly after they have been presented, and RECAPP-XPR collects data on when each stimulus was viewed. In Experiment 2, the study was replicated using the widely used image-sharing application Snapchat. A total of 197 participants were tested by 38 student experimenters, who manually presented the stimuli as “snaps” of experimentally controlled stimuli using the same experimental rates that had been used in Experiment 1. Like all snaps, these stimuli disappeared from view after a very short interval. In both experiments, we observed significant recall advantages for the first and last list items (primacy and recency effects, respectively), and there were clear tendencies to make more transitions at output between near-neighboring items, with a forward-ordered bias, consistent with temporal contiguity effects. The respective advantages and disadvantages of RECAPP-XPR and Snapchat as experimental software packages are discussed, as is the relationship between single-study-list smartphone experiments and long-term recency studies of real-world events.

The study of lists of verbal stimuli has been fundamental in shaping our understanding of how we encode and retrieve from human memory. Our textbooks, past and present, on the psychology of human memory (e.g., Baddeley, 1976, 1986; Baddeley, Eysenck, & Anderson, 2014; Crowder, 1976; Greene, 1992; Kahana, 2012; Murdock, 1974; Neath & Surprenant, 2003) are full of carefully controlled laboratory experiments in which the experimenter constructs lists of letters, digits, or words from particular stimulus sets, and following a specified retention interval, participants’ memory for the study list is tested by one of a variety of different testing methods. The vast majority of these studies have presented words at interstimulus intervals (ISIs) of one word every few seconds, with a relatively short retention interval (if any). Although numerous applications are capable of presenting and testing lists of verbal stimuli over short ISIs and short retention intervals in the laboratory, until recently it has been at best inconvenient, and at worst impractical, to present and test memory for lists of verbal stimuli in the real world over far longer interstimulus and retention intervals.

In this article, we compare and contrast the data and experimenter experience of two smartphone applications that can be used to conduct a straightforward but important long-term free recall study. These methods are a bespoke iPhone application called RECAPP-XPR and the widely used, multiplatform application Snapchat. The experiment we wished to conduct involved presenting many participants with a single list of eight words, with the words separated by an ISI of 1 h. An hour after presentation of the last word on the list, we asked participants to perform free recall—that is, to try to recall as many of the study words as possible, in any order that participants wished. The fundamental question that we wished to address was whether the benchmark findings that are observed in immediate free recall in the laboratory when the words are presented at a rate of one word every few seconds and tested immediately (such as the primacy, recency, and temporal contiguity effects) would also be observed when the words were presented and tested using far longer interstimulus and retention intervals.

In the sections that follow, we will describe the free recall task and consider two classes of theories that describe the most important empirical findings. We will then argue that these classes of theories can potentially be distinguished by considering the extent to which the benchmark findings observed at standard laboratory timescales can also be observed at far longer timescales. Finally, we will compare and contrast the two methods in some detail, and we will be able to compare and contrast the respective experimental results that we obtained.

## Free recall and the rationale for the proposed study

In a typical free recall trial, participants are presented with a list of words, one at a time, at a rate of one word every few seconds. Immediately after the presentation of the last list item, they must try to recall as many of the list items that they can, in any order that they wish. Two important empirical findings have emerged from studies using lists of unrelated words. First, participants tend to recall more words that are presented early in the list (a recall advantage known as the *primacy effect*) and more words that are presented late in the list (a recall advantage known as the *recency effect*) than items presented in the middle of the list (e.g., Deese, 1957; Jahnke 1965; Murdock, 1962). When recall performance is plotted by the position of each word on the experimenter’s list, this finding can be illustrated by the classic bowed or U-shaped serial position curve. Second, although participants are free to recall in any order, they nevertheless tend to initiate recall of a longer list with one of the last-presented list items (e.g., Deese & Kaufman, 1957; Hogan, 1975; Laming, 1999; but for shorter list lengths, see Ward, Tan, & Grenfell-Essam, 2010) and then to make successive recalls from near-neighboring list positions, with the highest tendency being to transition from one output to the next in forward serial order (Kahana, 1996), a finding known as the *temporal contiguity effect* (Healey & Kahana, 2014).

The serial position curve has been instrumental in the development of dual-store theories of free recall (e.g., Atkinson & Shiffrin, 1968, 1971; Raiijmaakers & Shiffrin, 1981). These theories assume that there are separate short-term and long-term memory stores (STS and LTS, respectively). The recency effect is assumed to reflect the direct output of the contents of the STS at test, whereas the primacy effect is assumed to be due to greater encoding of the early list items, which is due to additional rehearsal (e.g., Rundus, 1971) and/or a longer duration in the STS (e.g., Raiijmaakers & Shiffrin, 1981). Consistent with an STS explanation of the recency effect is the finding that a filled rehearsal-preventing interval of 15–30 s eliminates the recency effect (e.g., Glanzer & Cunitz, 1966; Postman & Phillips, 1965), while having little effect on the early and middle list items. By contrast, variables such as the list length (e.g., Murdock, 1962), the presentation rate (e.g., Glanzer & Cunitz, 1966), and different characteristics of the word pool, such as the word frequency (Sumby, 1963), have been shown to influence the early and middle list items, with little or no effect on the recency items. These findings have led some researchers to assume that the serial position curve can be characterized as comprising both short-term and long-term components (Glanzer, 1972). An STS interpretation of the temporal contiguity effect is also possible (Kahana, 1996). According to some dual-store accounts (e.g., Raiijmaakers & Shiffrin, 1981), items that are near neighbors in the list are more likely to co-reside in the STS during encoding, and so are more likely to be associated with each other in LTS. Therefore, when one item is recalled at test, the recalled item may help cue those neighboring items to which item–item associations have been strengthened during encoding.

One potential difficulty for an exclusively STS explanation of the recency effect is the finding of long-term recency effects: the observation that when participants are asked to free-recall real-world events, such as where they parked their car (da Costa Pinto & Baddeley, 1991), films they have seen at the cinema (Hitch & Ferguson, 1991), or their opponents in rugby matches (Baddeley & Hitch, 1977), they show enhanced recall of recent events, despite the fact that the events are separated by intervals of days and weeks, over which an STS interpretation would be untenable. Long-term recency effects are also observed in the free recall and cued recall of autobiographical memories that have been subjectively dated (e.g., Moreton & Ward, 2010; Rubin, 1982).

A second potential difficulty for an exclusively STS explanation of the recency effect and an exclusively STS explanation of temporal contiguity effects is that both temporal contiguity and recency effects are observed in the continuous-distractor free recall task. In this variant of free recall, a filled distractor interval is placed before and after every word in the list, including the last. When participants are asked to recall the presented words using this method, they recall more early and late list items, and they tend to transition during recall between near-neighboring items. It is common to use some mathematical problems as filler items (e.g., Bjork & Whitten, 1974; Howard & Kahana, 1999), but the filled delay between items can be the very same as that which eliminates the recency effect in immediate free recall (Tzeng, 1973). Thus, the primacy effect, the recency effect, and the temporal contiguity effect are observed in the continuous-distractor free recall task, using a method that renders an STS explanation unlikely.

One reaction to long-term recency effects and the continuous-distractor free recall data has been to propose new theories of free recall that assume both short-term and long-term recency mechanisms (e.g., Davelaar, Goshen-Gottstein, Ashkenazi, Haarmann, & Usher, 2005; Lehman & Malmberg, 2013; Raaijmaakers, 1993; Unsworth & Engle, 2007; Usher, Davelaar, Haarmann, & Goshen-Gottstein, 2008). An alternative reaction has been to abandon the distinction between STS and LTS and assume that recency effects and temporal contiguity effects are general properties of episodic memory that hold true over both the short and long term (Brown, Neath, & Chater, 2007; Howard & Kahana, 2002; Lohnas, Polyn, & Kahana, 2015; Neath & Brown, 2006; Polyn, Norman, & Kahana, 2009; Sederberg, Howard, & Kahana, 2008). Some researchers have further argued that principles of memory might be timescale invariant (Brown et al., 2007; Maylor, Chater, & Brown, 2001), or at least timescale similar (Moreton & Ward, 2010).

Regardless of which type of theory one is naturally drawn to, a clear and present need remains for data that can help us examine the serial position effects and temporal contiguity effects in long-term episodic memory using experimentally controlled stimuli presented over extended time periods. Without these data sets, it is difficult to test unitary memory predictions that the same memory mechanisms are responsible for the patterns of serial position and output order in both immediate memory and very long-term memory. Similarly, if different mechanisms are responsible for short-term and long-term memory, then there is a need to determine what basic benchmark findings should be accounted for by the proposed long-term memory mechanisms when experimentally controlled stimuli are presented in the absence of contributions from short-term memory.

One issue with the continuous-distractor free recall task is whether a filled delay really displaces all of the items from STS, or whether STS is merely attenuated by the filler activity. In the related field of working memory, it is common practice to perform complex span tasks (for reviews, see Camos & Barrouillet, 2014; Conway et al., 2005; Unsworth, Redick, Heitz, Broadway, & Engle, 2009), in which participants are required to encode lists of words for serial recall that are presented between successive filler activities (but for a different viewpoint, see Lehman & Malmberg, 2013). Despite the methodological similarities of the complex span task and the continual-distractor free recall task, it is widely assumed within the working memory literature that working memory is used to retrieve, rehearse, or refresh items that have not been in the focus of attention while the participants process the filler activities. This contrasts with the widely agreed hypothesis that the contents of short-term memory are permanently displaced by a filler activity. Moreover, although the interstimulus and retention intervals are increased in the continuous-distractor free recall task, these intervals are rarely greater than 15–30 s, which is perhaps enough to displace items from short-term memory, but hardly a strong test of whether benchmark findings in free recall can be observed across a wide range of timescales.

One issue with the studies showing long-term recency effects is that they have all used stimuli that are outside the experimenter’s control. In some cases the participants were responsible for generating their own stimuli. For example, the participant might have considerable say each day over where they might park their car (da Costa Pinto & Baddeley, 1991) or in the autobiographical events that they experience (Moreton & Ward, 2010; Rubin, 1982). In other cases, the participants are recalling from a series of semantically related events that occur in a prescribed order, such as the order of films at a cinema (Hitch & Ferguson, 1991) or their opponents in rugby matches (Baddeley & Hitch, 1977). These real-life event stimuli are clearly not selected randomly from an experimental stimulus pool. Moreover, participants often encode the real-life events incidentally, unaware that there will be a subsequent memory test, and events tend to be tested only once in tests of long-term recency. Although there are single-trial laboratory studies testing incidental learning, there are far more studies of free recall conducted using multiple study–tests under intentional learning conditions.

In summary, what is needed is new methods for presenting experimentally controlled verbal stimuli to participants over time periods of hours, days, and weeks, so that benchmark findings in long-term episodic memory can be established. It is self-evident that it is inconvenient at best, and impractical at worst, to invite participants into the laboratory to receive stimuli over such extended timescales. Fortunately, there may be increasing opportunities to make use of smartphone technologies to present and collect data (Miller, 2012), especially since smartphone ownership is increasingly prevalent (Smith, 2015).

## Using smartphone applications to study episodic memory over extended time periods

Recently, Cortis Mack, Cinel, Davies, Harding, and Ward (2017) pioneered the use of RECAPP,^1^ an iPhone application for presenting lists of words to participants’ smartphones at particular times. In three experiments, participants saw lists of between two and ten words each day (for between 10 and 50 days), and these words were selected at random without replacement from the Toronto Word Pool (Friendly, Franklin, Hoffman, & Rubin, 1982). Each day, the stimuli were presented at a rate of one word every hour, and one hour after presentation of the last word, participants were cued to recall all the words presented that day, responding by typing the words before submitting their responses. These experiments provide some of the best data to address whether the benchmark findings observed in immediate free recall are also observed when encoding and retrieval take place over far more extended time intervals. Cortis Mack et al. showed that there were clear temporal contiguity effects, but only the most shallow serial position curves using 1-h interstimulus and retention intervals. For example, in their Experiment 1, when the data from 40 participants tested with an eight-item list were aggregated over 10 days, recall at Serial Position 1 (.67) was significantly greater than recall at Serial Position 5 (.53), but the latter value did not differ from recall at Serial Position 8 (.62). These recall patterns were not affected when we manipulated the start times of the experimental list (Exp. 3), such that there were four different start and recall times.

When taken at face value, these aggregate data offer very little evidence supporting the claim that the benchmark serial position curves in immediate free recall in the laboratory are also observed for stimuli presented over far more extended timescales. This state of affairs is also disturbing if one compares the Cortis Mack et al. (2017) data with studies showing long-term recency (e.g., Baddeley & Hitch, 1977; da Costa Pinto & Baddeley, 1991). However, given that studies of long-term recency offer only a single test opportunity, one potential way to reconcile the two sets of data would be to consider the Cortis Mack et al. data on only the very first day of testing. When the serial position curve in free recall was limited to the first day of testing, there was far more pronounced bowing (the recall proportions at Serial Positions 1, 5, and 8 were .89, .46, and .68, respectively), but with only 40 participants, there was again a significant long-term primacy effect, but not a significant long-term recency effect.

Therefore, we wished to perform an experiment in which a large number of participants would be presented with a single list of eight words, with the words separated by an interstimulus interval of 1 h. An hour after the last word on the list, we then wished participants to perform free recall. This would allow us to determine whether there are reliable serial position effects in free recall on Day 1 of testing. Although a need would remain to integrate the standard laboratory free recall data with the aggregate Cortis Mack et al. (2017) data, obtaining reliable bowed serial position curves for Day 1 of testing with smartphones would reconcile the Cortis Mack et al. data with studies of long-term recency effects.

## The RECAPP-XPR application

The first smartphone application that we used was RECAPP-XPR, a modified version of an iPhone application, RECAPP, that we have been developing that has been used to study free recall, serial recall, and recognition memory over long timescales (Cortis Mack, et al., 2017; the open access source code is available at https://github.com/Recall-Project). RECAPP-XPR consists of (1) a mobile application and (2) a number of web and data storage components that integrate to support very long-term memory studies, experience-sampling studies, and longitudinal recall studies. RECAPP-XPR was originally designed to support mobile experience sampling and the deployment of context-sensitive questionnaires, but we have developed RECAPP-XPR to enable memory researchers to perform recall studies over long intervals. A number of system enhancements were necessary in order to address a number of key requirements to support memory studies.

The presentation requirements included (1) the ability to present stimuli randomly sampled without replacement from a chosen word pool, (2) the ability to vary the number of trials (or lists) in a survey (or experiment) and the number of stimuli presented in a trial (i.e., the list length), (3) the ability to manipulate the ISI between consistent temporal triggers, (4) the ability to manipulate the duration over which stimuli will remain available to be viewed, and (5) the ability to modify an orienting question that guides how participants engage with the stimuli, using a Likert scale (a capability that was already in the system). The response requirements for complete and accurate data collection included (1) the ability for participants to perform Likert ratings within a specified time frame and (2) the ability for participants to be able to use free text to enable recall within a specified time frame. Finally, the experimenter requirements for efficient data management included (1) the ability for the experimenter to download a complete schedule of stimulus allocations across participants, so that it is easy to identify the serial order in which the words were presented to the participants; (2) the ability for the experimenter to access and download the participants’ responses to the Likert questions; and (3) the ability for the experimenter to access and download the participants’ responses to the recalled items.

## The RECAPP-XPR architecture

**Fig. 1:**
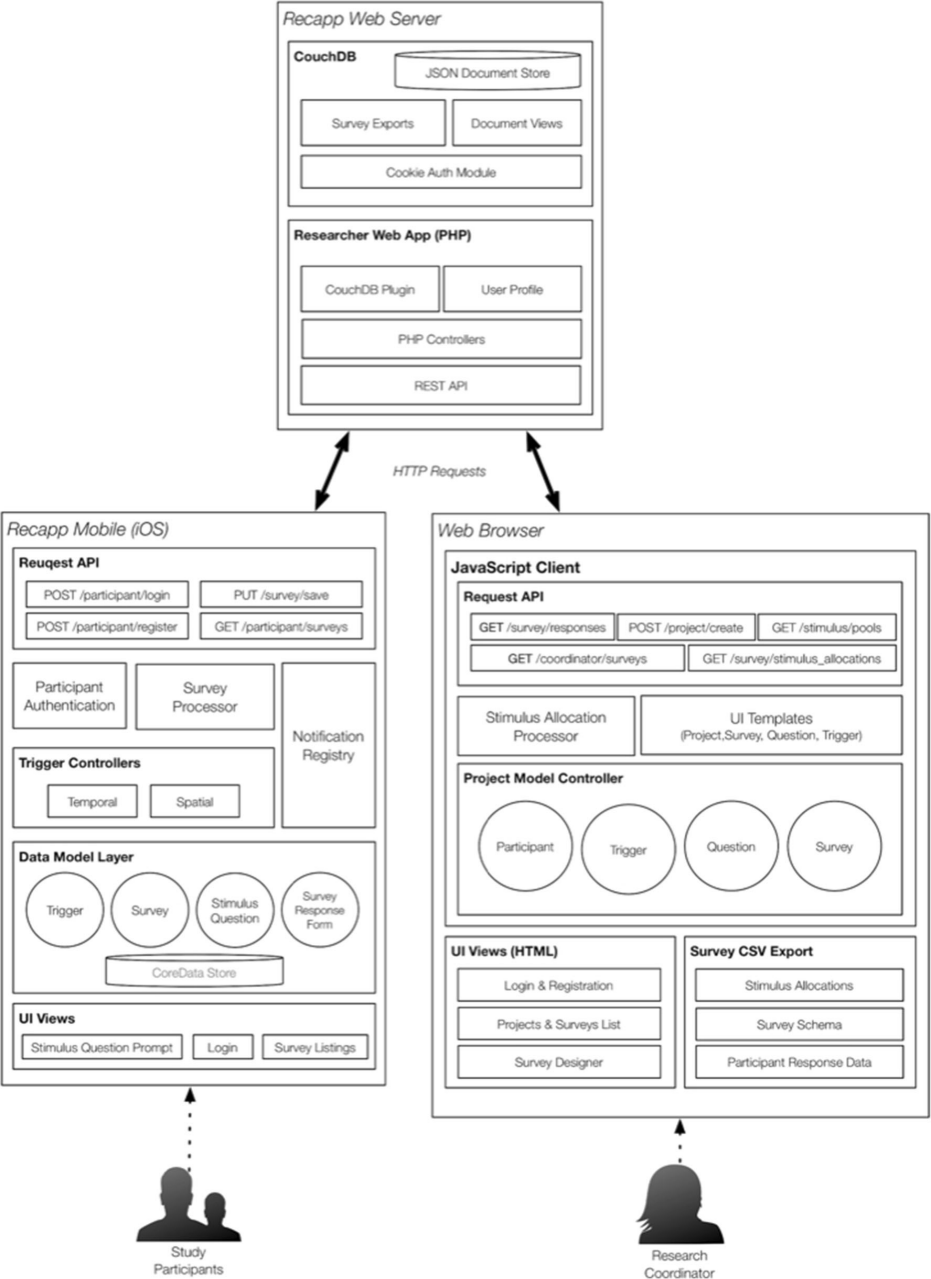
RECAPP-XPR high-level architectural system

Figure 1 illustrates the core technical components of the RECAPP-XPR system and outlines how the system supports researchers in designing studies and supports participants in responding to memory recall surveys.

The design and deployment of memory experiments is implemented through a series of distributed technical components, which include (1) a web interface that enables experimenters to construct and configure memory surveys in a managed and intuitive manner and (2) a centralized web service that stores information pertaining to the configuration of the experiment and disseminates this to participants’ mobile devices through a supporting application programming interface (API) based on the Representational State Transfer (REST) architecture.

## Setting up RECAPP-XPR: Projects, surveys, triggers, and questions

The implementation of a multitrial experiment in RECAPP-XPR is a straightforward process that takes only about 5 to 10 min, depending on the number of participants that need to be added. In the following paragraphs we detail the RECAPP-XPR framework, discuss the web management interface from the point of view of the experimenter, and summarize the technical details and procedures.

In the RECAPP-XPR framework, a multitrial memory experiment is known as a project, and a memory trial (or list) is known as a survey. Projects and surveys have a one-to-many mapping, so that a set of participants can easily be allocated to a multitrial experiment, each trial consisting of multiple stimuli. A survey itself contains two core elements: (1) a set of prompts to participants that display the stimuli and query a participant for a response, and (2) the trigger that dictates when the stimuli and response request will be displayed. Each trigger is attached to a single survey and is based on a specified time and/or location. Although RECAPP-XPR supports both questionnaire and stimulus projects, for the purposes of this article we will focus on stimulus-based studies within RECAPP-XPR.

**Fig. 2:**
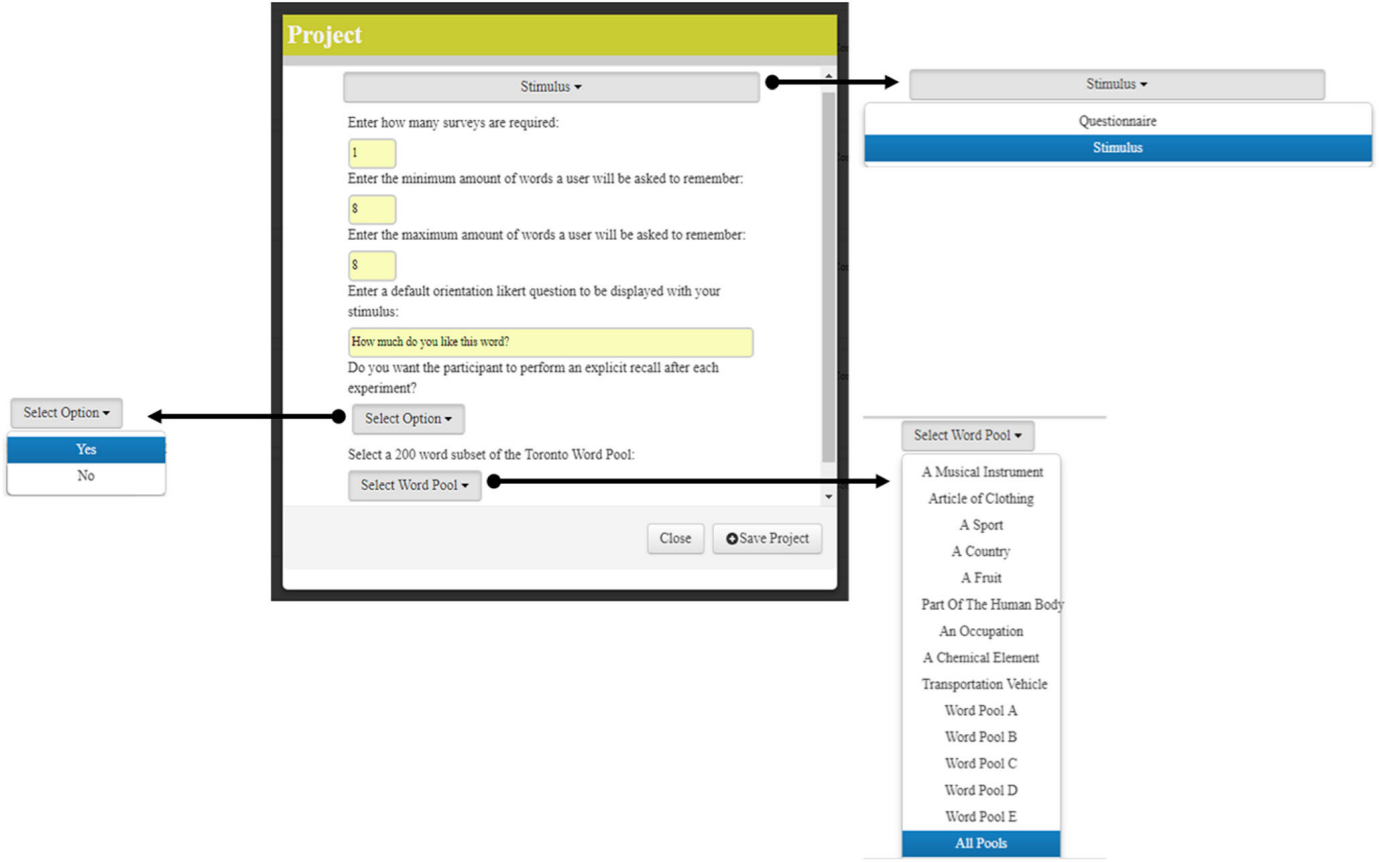
Setting up a list-learning experiment in the RECAPP-XPR web-based interface

Figure 2 shows the web management interface to set up a list-learning experiment within RECAPP-XPR, including all options and drop-down menus. On creation of a new project, the experimenter is guided through an interactive wizard to configure a number of experimental parameters. The interface requests that the experimenter enter the number of surveys (i.e., trials or lists), the minimum and maximum numbers of words per trial, as well as an orientation question of choice. Experimenters also can choose whether they want participants to have a recall test at the end of the trial and can select the word pools (e.g., Toronto Word Pool; Friendly et al., 1982) from which the to-be-presented words are to be sampled. Once all these preferences are inputted, the experimental structure is complete (for a detailed explanation of how the experimental structure is processed, see the Appendix, Note 1).

**Fig. 3:**
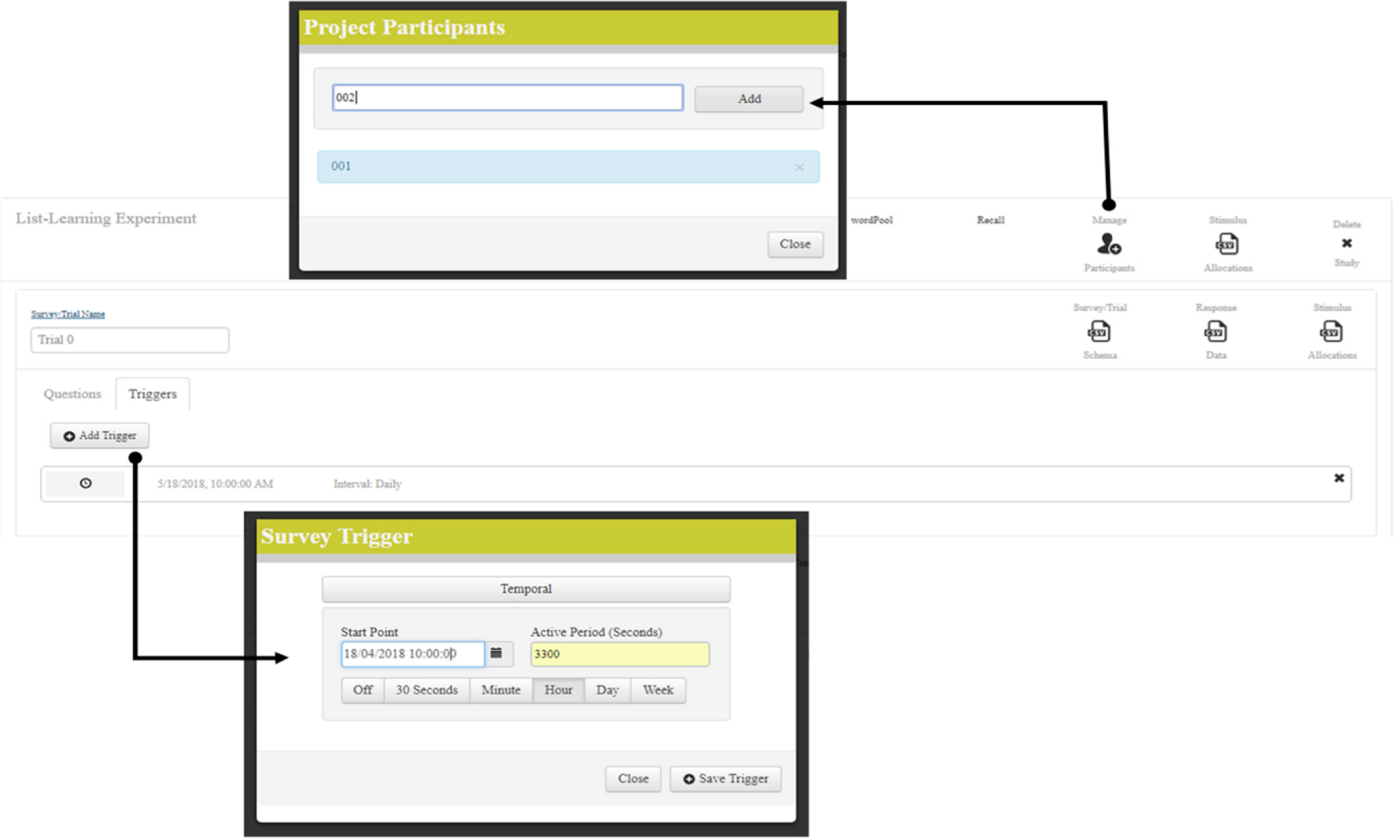
Adding participants and temporal triggers to a list-learning experiment in the RECAPP-XPR web-based interface

Once the experimental structure is complete, the experimenter must select either a spatial or a temporal trigger for the experiment to start and then add participants to the experiment. Figure 3 shows the web interface, which allows the experimenter to delete the study, add participants, and add triggers, as well as to access stimulus allocation and response files.

In setting up a temporal trigger, a series of mandatory fields must be specified, which include (1) a timestamp to indicate the start date and time of the experiment, (2) an “active period” that represents the duration (in seconds) for which each stimulus can be accessed via the mobile application, and (3) the rate at which the trials are to be presented (from among five options: every 30 s, minute, hour, day, or week).

In our experiments to date, we have only utilized the temporal trigger function, but the spatial trigger option is functional, albeit in need of more development. As it stands, the iOS device running RECAPP-XPR needs to access users’ GPS coordinates; however, the participants’ location data are not stored or handled by the RECAPP-XPR system, since they are only used within the device. Consequently, surveys and stimuli can be presented to participants when they enter a particular geographical area that is predefined through the spatial trigger configuration menu on the RECAPP-XPR website. Furthermore, the location accuracy is dependent on the iOS platform’s capability, which varies depending on the method of localization (i.e., cell towers, WiFi, or GPS) available at a specific point in time. For this reason, accuracy can range from 8 to 600 m.

**Fig. 4:**
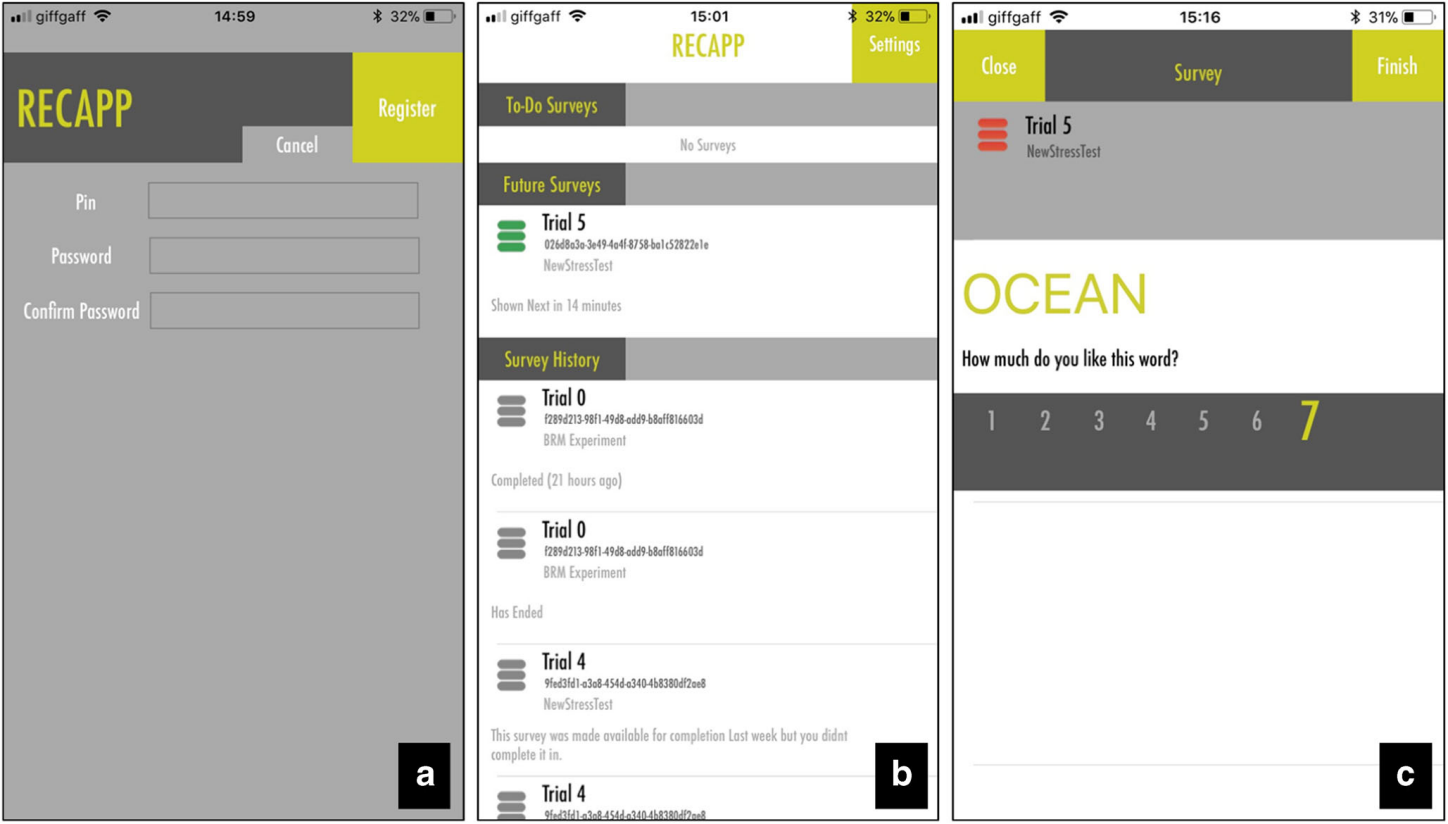
Screenshots of the RECAPP-XPR iPhone application interface. (A) Registration screen. (B) Schedule for current, future, and past trials. (C) Stimulus presentation, coupled with an orientation question and Likert scale

Once a project and the associated surveys have been configured, stimuli are allocated whenever a new participant is added to the project. To add participants to the study, the experimenter simply needs to add a participant PIN in the “Manage Participants” input box. The participant PINs are made up exclusively of digits and can be added prior to recruiting participants. The participant can then be provided by the experimenter with a mandatory PIN and password during the mobile registration process (Fig. 4A). The use of a PIN acts as a user identifier that ensures that all user data managed through RECAPP-XPR are stored anonymously, safeguarding user privacy and mitigating the risk of an attacker linking survey response data to an individual.

After each participant has been added, the stimulus allocation module (see Fig. 1) first queries the RECAPP-XPR web server REST API and downloads the specified word pool, which is stored as a JSON array document in the CouchDB database (see the Appendix, Note 2). Second, the module processes the specified details of each survey, randomly allocates the participants’ stimuli, and marks each assigned stimulus so that for each participant, the words are sampled without replacement.

## In-situ study participation using RECAPP-XPR

After configuration of the memory study, participants can log in to the RECAPP-XPR mobile application via an iOS device and can automatically download all survey information (see Fig. 4B). To notify participants when new stimuli and recall questions are available to view, each trigger interval is registered as an iOS local banner notification. The application is able to circumvent limitations to the maximum number of allowable registered iOS user interface (UI) local notifications (limited to 64 per application) by only registering notifications for triggers scheduled within a 12-h window.

Once a participant signs in, the application automatically queries the RECAPP-XPR web service for available projects configured with the participant’s PIN identifier (see the Appendix, Note 3, for technical details). First, the survey, trigger, and stimulus question configurations are identified and stored as native iOS Core Data objects. Second, each survey’s associated temporal triggers are assessed, to determine whether the survey is expired, completed, active, or available. Depending on each survey’s status, they are displayed to the user across three sections (“To-Do Surveys,” “Future Surveys,” and “Survey History”) within a list view (shown in Fig. 4B).

The “To-Do Surveys” section displays surveys that are not yet completed. A survey will only remain in this section until either a user responds or the “active period” has expired. “Future Surveys” represent surveys that are active, with a pending stimulus question to be made available at some time in the future. Finally, the “Survey History” section displays surveys that either have been completed or have expired without a response. Similar to mobile social-networking applications (e.g., Facebook), a pull-down refresh capability was included in order to enable participants to override the automated periodic caching (every 20 s) and force the mobile application to synchronize with the RECAPP-XPR server in order to cache new survey configurations and update the survey listings, based on the current time. A stimulus survey can be configured to contain two types of question: a stimulus question, which comprises an allocated stimulus word combined with an orientation Likert question, and a recall question, which provides a participant with a text field in which to input as many of the previous stimuli displayed as the participant can recall. A recall question is optional and can be set by the research coordinator, depending on the type of memory experiment to be supported through RECAPP-XPR.Once the pre-registered temporal triggers are met, participants receive a notification on their device informing them that a new stimulus is available. Upon clicking the notification (or selecting from the “To-Do Survey” listing in the UI), participants are presented with a stimulus, coupled with an orientation question via a modal view (Fig. 4C). Survey responses to Likert orientation questions and words submitted through a recall question are collated locally on the device in a “Survey Response Form” Core Data object. This object is then converted and saved to the web service document store for study monitoring and reporting.

Experimenters can monitor ongoing project progress and export survey response data as downloadable comma-separated value (CSV) files via the project management web interface. The web component supports the exporting of three forms of data related to each survey in a project, including (1) schemas that describe each survey, such as the number of stimulus questions posed to participants, (2) the stimulus allocated to each participant associated with a survey, and (3) the response data to the stimulus orientation questions and words provided by participants when asked to perform an end-of-survey recall task.

## The Snapchat application

We contrast our experiences and our obtained recall data using RECAPP-XPR with a similar experiment performed using Snapchat, a widely used, time-limited instant-messaging application that is freely available for both the Android and iOS operating systems. As of February 2018, Snapchat had 187 million daily users (Statista, 2018), and it is increasingly used by teenagers to share “selfies” and photos embedded with text and doodles. According to Statista, there were an estimated 10 billion Snapchat-generated mobile video views per day in May 2016. Users consider snaps to be an easy and fun way of communicating with close friends and family via their smartphone (Piwek & Joinson, 2016). One important feature of Snapchat is that pictures and videos are available only for a short time (between 1 and 10 s; the viewing time is selected by the sender) before they disappear from the receiver’s phone. To play, the receiver must maintain contact with their smartphone’s touchscreen, reducing the opportunity to take a screenshot or to operate an additional camera (Piwek & Joinson, 2016). Moreover, the sender is informed if a screenshot is taken. The sent picture or video also vanishes from the sender’s phone. The application therefore has the potential to present information to participants’ smartphones at any chosen interval, and unlike emails and texts, the presented material cannot easily be repeatedly reviewed.

## A comparison of RECAPP-XPR and Snapchat

In this section, we summarize the main similarities and differences between our two smartphone applications. First, considering the application itself, Snapchat is already widely installed on a large number of undergraduate participants’ smartphones, and it operates on both iOS and Android platforms. Most users will already be highly familiar and very proficient in its operation and will have a readymade list of potential participants in their contact list. By contrast, RECAPP-XPR is an iPhone app that, because it is still in development, is only available for a 90-day period by invitation from the beta iOS apps store, TestFlight.

Second, from the participants’ perspective, both applications present experimenter-controlled stimuli to a remote mobile device, allowing experimenters to present participants with stimuli to encode throughout the day, obviating the need for participants to come into the laboratory. Moreover, both applications display the stimuli for a limited period, either an interval set by Snapchat sender (1–10 s) or until the RECAPP-XPR participant responds with a Likert value. Although it is possible to take screenshots of both applications, senders are notified when using Snapchat. RECAPP-XPR, but not Snapchat, allows the experimenter to set a limited time window during which the stimulus is available to view. RECAPP-XPR, but not Snapchat, allows the experimenter to set up a spatial trigger (which can be used with or without a temporal trigger), whereby a stimulus will be presented when the smartphone arrives within a specified distance from a designated location.

Third, from the experimenter’s perspective, RECAPP-XPR allows an experiment to be designed and set up in 5 min, after which the data presentation and data collection is fully automated. In RECAPP-XPR, data are collected on Likert responses, providing the experimenter with the timings of user engagement with the different stimuli. The recall data are also collected and sent as a CSV file that can be easily sorted and compiled into a results file. By contrast, stimulus presentation by the experimenter in Snapchat is labor-intensive. The images of the experimental stimuli in Snapchat must be manually snapped using printouts of verbal stimuli via the smartphone camera. In Snapchat, the experimenter must also set external alarms to alert and notify the experimenter for each presentation, and each stimulus presentation requires the prepared printout of the stimulus, and a short manual series of button presses allows the snapped image to be shared with each participant.

## Experiment 1

In Experiment 1, 76 participants were presented with eight unrelated words drawn at random from the Toronto word pool. Critically, the words were presented at a rate of one word every hour, and the free-recall test was administered 1 h after presentation of the last item. These timings made it impractical for the stimuli to be presented in the laboratory. The main purpose of the experiment was to determine whether benchmark findings of the primacy effect, the recency effect, and the temporal contiguity effect that are observed with short timescales and immediate testing are also present at much longer timescales. A single study–test trial was used because classic studies that have shown long-term recency effects have used a single test. Furthermore, Cortis Mack et al. (2017) showed that when presenting participants with a list of words (at a rate of one every hour) every day for between 10 and 50 days, the aggregate serial position curves were mostly shallow. However, when analyzing the data from the first day of testing only, these Day 1 serial position curves appeared more bowed than the overall aggregate curves. Unfortunately, in the Cortis Mack et al. experiment, there were insufficient Day 1 data to determine whether such trends were reliable. This issue was circumvented in Experiment 1 by testing almost twice as many participants as Cortis Mack et al. had.

Using RECAPP-XPR, the setup of the experiment took no more than 5 min, plus the time taken to type in and upload the participants’ phone details in a single CSV file. Once the experimenter had set up the experiment and briefed the participants, all of the stimulus presentations and data collection occurred automatically.

### Method

#### Participants

A total number of 76 students from the University of Essex participated in exchange for a £5 payment. To be included in the study, participants had to possess and be a regular user of an Apple iPhone 6 or later model, running operating system iOS 8.0 or later.

#### Materials and equipment

The total stimulus set consisted of 1,000 words taken from the Toronto Word Pool (Friendly et al., 1982). Each word was presented in uppercase font on participants’ iPhone screens using the application RECAPP-XPR.

#### Design

The experiment used a within-subjects design. There was one independent variable: serial position, with eight levels. The main dependent variable was the proportion of words recalled correctly.

#### Procedure

Each participant attended the laboratory for an initial briefing, during which the experimenter ensured that the RECAPP-XPR application was properly installed on their iPhone and familiarized the participant with the application and the task itself. This initial briefing took place between one and two days before the list was presented on participants’ iPhones. The stimuli for each participant were eight randomly selected words from the Toronto Word Pool. Each word was presented in uppercase font and was left-justified on participants’ iPhones using the RECAPP-XPR application.

On the study day, participants received the first phone notification from RECAPP-XPR at 10:00 a.m., informing them that a new stimulus was available. Each stimulus was available for 55 min after the notification. Upon tapping the notification, participants were presented with a single to-be-remembered word, below which was a pleasantness-rating question. Participants were asked to remember the word for a later test and to rate the pleasantness of each word on a 7-point Likert scale. Having selected their pleasantness rating, the participants were asked to press “Finish” at the top right corner of the screen, after which the word could no longer be viewed. The next stimulus was presented at 11:00 and this continued for the eight stimulus presentations.

One hour after the last list item had been presented (i.e., at 18:00), the participants were prompted by a RECAPP-XPR phone notification to enter as many words as they could in any order that they liked within a small textbox within RECAPP-XPR. Once they were satisfied that they had typed in as many words as they could remember, participants were required to press “Finish.” During the briefing, participants had been told not to write any of the words down as the words were presented, and they had also been advised that should they miss the recall period, they could manually send their recalls to the experimenter via email or text message.

### Results

#### Missing data

Five out of the 76 participants did not interact with the application during this experiment, and since no data were available from these participants, they were excluded from the subsequent analysis. Furthermore, on a number of occasions, participants did not view and rate all of the words presented by RECAPP-XPR within the 55-min time limit, and a total of eight participants missed the 55-min recall period, and therefore recalled manually via a direct email or text message to the experimenter (these data were not excluded). In the analyses reported below, we examined the recall of 504 out of 568 words (88.7% of the total presented words) that had been viewed across 71 participants.

#### Serial position curves

**Fig. 5:**
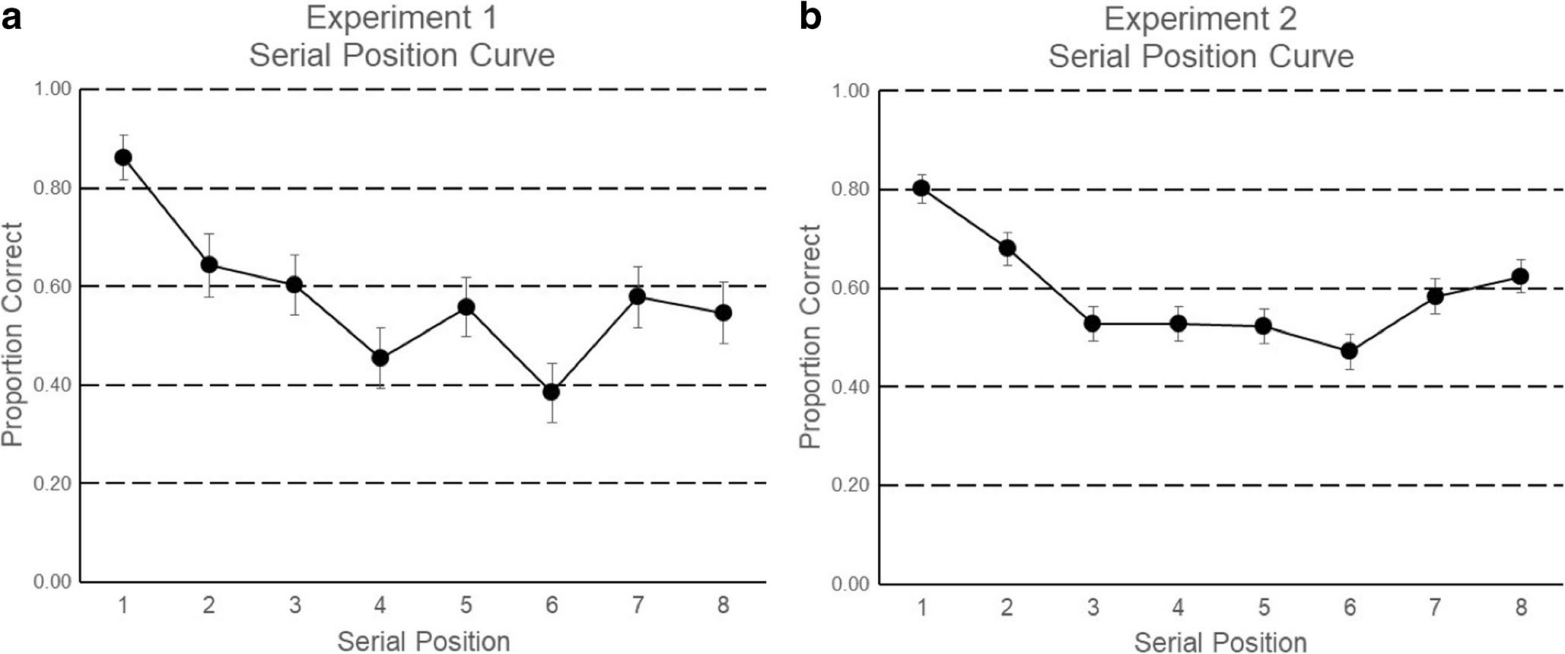
Serial position curves from Experiment 1 (A) and Experiment 2 (B)

Figure 5A shows the proportion of viewed words that were recalled correctly as a function of their serial position. To test for primacy and recency effects, we compared the recall at the start (i.e., Serial Position 1) and the end (i.e., Serial Position 8) of the list to the lowest-recalled serial position in the list, which was Serial Position 6. The number of participants who correctly recalled Serial Position 1 but did not recall Serial Position 6 was 27, whereas only two participants showed the reverse pattern. There were 25 tied scores, representing 20 participants who recalled both Serial Positions 1 and 6, along with five participants who recalled neither of the items. A related-samples McNemar test confirmed that recall was significantly greater in Serial Position 1 than in Serial Position 6 (*p* < .001), therefore showing a significant primacy effect. The number of participants who correctly recalled Serial Position 8 but not Serial Position 6 was 17, whereas six showed the reverse pattern. There were 35 tied scores, representing 15 participants who recalled both Serial Positions 6 and 8, as well as 20 participants who recalled neither of the items. A related-samples McNemar test confirmed that the recall in Serial Position 8 was significantly greater than that in Serial Position 6 (*p = .*035), therefore showing a significant recency effect.

#### Output order

Table 1 shows the number of list items recalled across the different output positions. Although participants were asked to recall as many words as they could in any order they liked, participants were much more likely to start their output with the first item on the list (29 out of 68 valid responses; 42.6%) than to start with the last item on the list (ten out of 68 valid responses; 14.7%).

**Table 1.** Data from Experiment 1: Distribution of words recalled as a function of serial position and output position

Serial Position	Output Position								No Response	Unseen Words
	1	2	3	4	5	6	7	8		
SP1	**29**	5	8	3	3	1	1	0	8	13
SP2	2	**23**	3	5	1	2	0	0	20	15
SP3	10	8	**12**	3	4	0	0	1	25	8
SP4	3	6	5	**9**	4	1	2	0	36	5
SP5	9	5	7	4	**8**	5	0	0	30	3
SP6	2	2	8	5	2	**5**	1	0	40	6
SP7	3	10	4	8	5	1	**5**	1	27	7
SP8	10	3	5	5	2	5	3	**2**	29	7

“No Response” refers to words that were not recalled because participants finished their recall, and thus did not produce any further responses. “Unseen Words” refers to those words that were not viewed by the participants within the allocated 55 min, and therefore were missed. The values in bold indicate responses in which the participants outputted a word in the same order as it had been presented, despite this not having been a task requirement

**Fig. 6:**
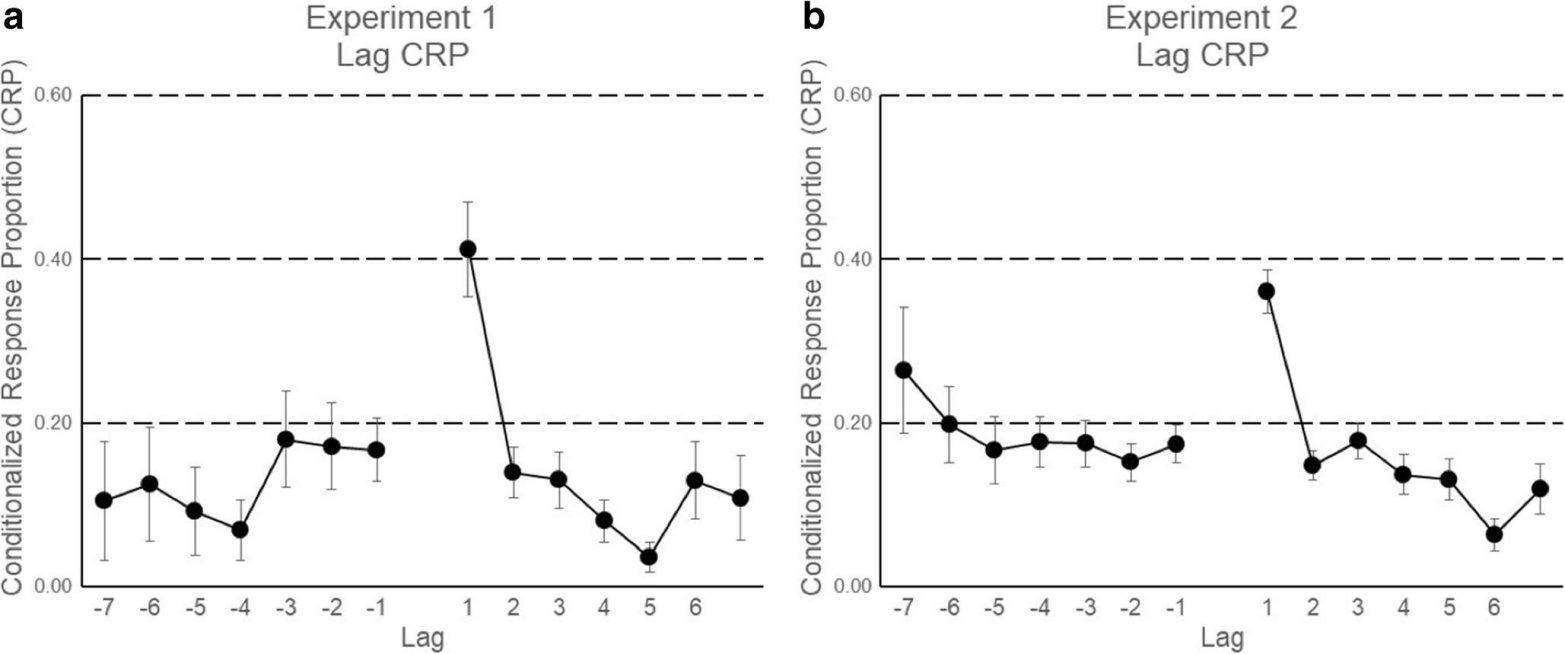
Conditionalized response probabilities (CRPs) for each lag in Experiment 1 (A) and Experiment 2 (B)

Table 2 shows the transitions between consecutive words recalled. It is of particular interest that participants preferred to output consecutive items on the list in succession, such that the output *n*+1 was often the word at the subsequent serial position from output *n* (see the values in bold in the table). These transitions can also be used to calculate the lag (Kahana, 1996), by first subtracting the serial position of the word recalled in output position *n* from that of output *n*+1. Figure 6A shows the lag transitions of Experiment 1, conditionalized by the number of opportunities to make these transitions at various lags (*conditionalized response probabilities*; CRPs).

**Table 2.** Data from Experiment 1: Distribution of transitions of successive pairs of responses (items *n* and *n*+1)

Serial Position of Output Position *n*	Serial Position of Subsequent Item (Output Position *n*+1)								Error	No Response
	1	2	3	4	5	6	7	8		
1	–	**18**	5	3	3	0	6	5	4	5
2	4	–	**15**	4	2	3	3	1	2	2
3	3	4	–	**9**	6	2	2	1	6	4
4	4	3	2	–	**5**	2	3	2	3	5
5	2	1	1	3	–	**8**	3	4	6	9
6	2	1	2	1	0	–	**10**	3	0	6
7	3	1	1	4	7	4	–	**5**	3	7
8	2	3	2	1	2	3	6	–	2	12
Error	1	3	0	2	4	1	1	4	7	9
No response	–	–	–	–	–	–	–	–	–	191

“No Response” refers to the point at which the participant, having finished recall on a given trial, did not produce any further responses. The values in bold indicate those successive responses in which participants transitioned between subsequent items on the list

The lag-CRP curve in Fig. 6A shows that the data observed with words presented at a rate of one word/hour resemble those obtained with faster presentation rates: There is a strong tendency to transition between nearby serial positions (small absolute values of lag), with an asymmetric bias to transition in forward order (e.g., lag +1 greater than lag –1).

### Discussion

Experiment 1 showcased the use of RECAPP-XPR to present stimuli and test recall of a single list of eight words presented at a rate of one word every hour and a retention interval of 1 h, a presentation schedule that would be impractical in the laboratory. We wished to highlight the ease with which these data could be collected, once the RECAPP-XPR application had been installed on participants’ iPhones. Using the web-based interface, a new experiment took less than 10 min to set up, after which the stimuli were randomly allocated to each participant, and the presentation and data collection were triggered entirely automatically. All that was required was for the collected data to be concatenated into a single spreadsheet, and the data could be easily sorted by participant, list, and serial position (or output order) for further analyses.

The data clearly show that participants recalled the first word and the last word better than the lowest serial position (Serial Position 6). They also showed a clear tendency to transition in their recalls between words that were successively presented (lag +1; Kahana, 1996). Therefore, in this straightforward test of long-term episodic memory, we found significant primacy and recency effects, as well as a clear temporal contiguity effect.

Primacy and recency effects had been obtained on Day 1 of testing in Cortis Mack et al. (2017), but unfortunately, with less than 40 participants contributing to the Day 1 serial position curve, there had been insufficient statistical power to confirm a significant recency effect. In this replication with almost double the number of participants, we were able to confirm significant long-term primacy and recency effects in a long-term test of episodic memory, suggesting that there are broad similarities between the data patterns in immediate recall and long-term testing (at least for Day 1 of testing). Our findings also help reconcile the Cortis Mack et al. data with real-world long-term recency effect studies: Now both types of studies show primacy and recency with a single study–test list. We will continue with our discussion after we have reported a replication using Snapchat.

## Experiment 2

In Experiment 2, 197 participants were presented with a single list of eight unrelated words, drawn at random from the Toronto Word Pool, using the highly popular multimedia application Snapchat. Again, the experimental stimuli were presented at a rate of approximately one word every hour, and the free recall test was administered approximately 1 h after presentation of the last item.

The Snapchat application was highly familiar to the experimenter and participants. However, the stimulus presentation was entirely manual, requiring the capturing and sending of a photographic image of the designated stimulus word at the designated time. The workload and coordination necessary to send almost 1,600 stimuli, selected at random from 1,000 possible words, to almost 200 participants prohibited the task being undertaken by a single experimenter. Rather, we recruited 38 student experimenters, who each delivered the stimuli to a subset of participants.

The main purpose of the experiment was again to determine whether benchmark findings of the primacy effect, the recency effect, and the temporal contiguity effect that have been observed with short timescales and immediate testing are also present at much longer timescales. The experiment also served to validate the findings of Experiment 1, and the differences between the methods and data provided useful comparisons.

### Method

#### Participants

A total of 197 participants were recruited by 38 student experimenters as part of their undergraduate coursework. To be included in the study, participants had to possess a smartphone and be a regular user of the application Snapchat.

#### Materials and equipment

The stimuli were identical to those of Experiment 1. Each word was presented via a “snap,” whereby participants saw a photographic image of a word in uppercase font via the Snapchat application on their smartphone.

#### Design

The design of Experiment 2 was identical to that of Experiment 1.

#### Procedure

Each student experimenter was responsible for recruiting a minimum of four participants who were Snapchat users. Each student experimenter was randomly allocated a word list that consisted of eight randomly selected words from the Toronto Word Pool (Friendly et al., 1982). All of the participants recruited by a particular student experimenter saw the same words in the same order. To maintain consistency, experimenters were given the instructions to relay to their participants, and all words on the list were printed in black on white paper in uppercase 70-point font.

Once participants had been recruited, they were informed that on a day mutually agreed upon with the student experimenter, they would be sent eight snaps presented at a rate of one every hour. The snap consisted of a photo of a printed word sent separately to each tested individual, and once a word was opened, participants could view it for a maximum of 10 s. Participants were instructed to try to remember the word for a later test and to answer how often they had said, heard, or read the presented word, on a 3-point scale (1 = *never*, 2 = *once*, 3 = *more than once*). Their response was sent via Snapchat, and the student experimenter manually recorded them. The next stimulus was presented in the following hour on the hour. An hour after the last list item had been presented, participants were asked to type in as many words as they could remember in any order they liked. Participants could recall via Snapchat chat or via text message, which they sent to the student experimenter once they had finished their recall.

### Results

#### Missing data

Since Snapchat only restricts the availability of an unopened stimulus once it exceeds 30 days, there were no missing data in this experiment. However, this comes at the expense of strict presentation rates, such that in this experiment the participants could technically view the stimuli after the 55 min of allocated time were up.

#### Serial position curves

The proportions of correctly recalled words are shown in Fig. 5B. As in Experiment 1, we compared the recall at the start (i.e., Serial Position 1) and the end (i.e., Serial Position 8) of the list to the lowest-recalled serial position in the list, which was Serial Position 6. The number of participants who correctly recalled Serial Position 1 but did not recall Serial Position 6 was 74, whereas only nine participants showed the reverse pattern. There were 114 tied scores, representing 84 participants who recalled both Serial Positions 1 and 6, as well as 30 participants who recalled neither of the items. A related-samples McNemar test confirmed that recall was significantly greater in Serial Position 1 than in Serial Position 6 (*p* < .001), therefore showing a significant primacy effect. The number of participants who correctly recalled Serial Position 8 but not Serial Position 6 was 63, whereas 33 showed the reverse pattern. There were 101 tied scores, representing 60 participants who recalled both Serial Positions 6 and 8, along with 41 participants who recalled neither of the items. A related-samples McNemar test confirmed that recall was significantly greater in Serial Position 8 than in Serial Position 6 (*p = .*003), therefore also showing a significant recency effect.

#### Output order

Table 3 shows the numbers of list items recalled across the different output positions. Consistent with Experiment 1, participants were much more likely to start their output with the first item on the list (108 out of 197 possible responses, or 54.8%) than to start with the last item on the list (22 out of 197 responses, 11.2%).

**Table 3:** Data from Experiment 2: Distribution of words recalled as a function of serial position and output position

Serial Position	Output Position								No Response
	1	2	3	4	5	6	7	8	
SP1	**108**	13	19	12	4	1	0	1	39
SP2	16	**71**	20	9	11	3	2	0	65
SP3	3	16	**43**	21	10	8	3	0	93
SP4	9	20	22	**31**	12	5	3	0	95
SP5	8	15	19	21	**28**	9	3	0	94
SP6	11	10	12	14	17	**23**	4	2	104
SP7	14	16	15	24	21	9	**14**	2	82
SP8	22	19	19	15	9	14	9	**15**	75

“No Response” refers to the point at which the participant, having finished recall on a given trial, did not produce any further responses. The values in bold indicate responses in which the participants outputted a word in the same order as it had been presented, despite this not having been a task requirement

Table 4 shows the transitions between consecutive words recalled. Consistent with Experiment 1, participants preferred to output consecutive items on the list in succession, such that the output *n*+1 often held the subsequent serial position from output *n* (see the values in bold in the table). The lag-CRP curve in Fig. 6B shows a strong tendency to transition between nearby serial positions (small absolute values of lags), with an asymmetric bias to transition in forward order (e.g., lag +1 greater than lag –1).

**Table 4:** Data from Experiment 2: Distribution of transitions of successive pairs of responses (items *n* and *n*+1)

Serial Position of Output Position *n*	Serial Position of Subsequent Item (Output Position *n*+1)								Error	No Response
	1	2	3	4	5	6	7	8		
1	–	**67**	12	20	8	9	4	14	9	15
2	9	–	**44**	13	14	8	12	9	4	19
3	4	3	–	**29**	14	8	9	6	4	27
4	10	9	8	–	**27**	13	11	9	4	11
5	4	11	10	10	–	**24**	13	10	1	20
6	6	7	6	4	5	–	**30**	10	1	24
7	7	7	11	6	11	10	–	**38**	2	23
8	9	8	4	10	8	5	19	–	2	57
Error	1	4	6	1	8	5	3	4	5	0
No response	–	–	–	–	–	–	–	–	–	610

“No Response” refers to the point at which the participant, having finished recall on a given trial, did not produce any further responses. The values in bold indicate those successive responses in which participants transitioned between subsequent items on the list

### Discussion

Experiment 2 essentially replicated the findings from Experiment 1 using a far more labor-intensive method. The main advantage of Snapchat is its heightened availability: It is already highly familiar to the majority of participants of student age, and it is freely downloadable on both Android and iOS smartphones. The main disadvantage is that presentation of the stimuli is entirely reliant on the experimenter manually selecting the appropriate stimulus for each participant every hour. With 197 participants, this required the sending of almost 1,576 separate images of the target stimuli. It should be noted that the method using Snapchat also required the manual request and collation of 197 separate participants’ recalls.

Despite the procedural differences, it is nonetheless reassuring that the patterns of data are broadly consistent with those found in Experiment 1. Specifically, we again observed significant primacy and recency effects, and we showed that there was a clear tendency to initiate recall with the first list item and to recall in forward order.

## General discussion

There were two main aims of the present experiments. The main theoretical purpose of our studies was to determine whether we might find evidence for three benchmark findings of immediate free recall (primacy effects, recency effects, and temporal contiguity effects) when we examined the free recall of experimentally controlled events presented over greatly extended temporal schedules. The main methodological aim from our studies was to compare and contrast the strengths and weaknesses of two smartphone applications for the presentation and recall of a list of words presented at a rate of 1 word every hour. The two methods were (1) our bespoke iPhone app, RECAPP-XPR, and (2) the social media app Snapchat.

### Theoretical contribution from empirical findings

We consider first our main empirical findings: we found evidence for all three benchmarks findings in long-term free recall using experimentally controlled word lists that were presented with ISIs and retention intervals of 1 h. Thus, when participants were presented with a list of eight words presented throughout the day, they recalled more words from the beginning of the list (primacy effect) and more words from the end of the list (recency effect) than the late-middle list items. Moreover, there was a clear tendency to transition between near-neighboring items, with a particular bias to transition in a forward direction—that is, to recall item *n*+1 immediate after the recall of item *n*.

These findings greatly strengthen a tentative suggestion in the Cortis Mack et al. (2017) article, that long-term serial position curves for unrelated items could be obtained in a free recall test of the very first list, when participants are most likely to be free from proactive interference, and most likely to use the smartphone application as an effective and specific cue to recall the presented list items. These findings help reconcile the long-term free recall of unrelated items with studies that have used a single test of real-world phenomena. These showed clear long-term recency effects for car parking events (da Costa Pinto & Baddeley, 1991), rugby opponents (Baddeley & Hitch, 1977), autobiographical events (Moreton & Ward, 2010; Rubin, 1982), and films seen at the cinema (Hitch & Ferguson, 1991). These findings also replicate the long-term temporal contiguity effects that were observed in the aggregate data from the multitrial Cortis Mack et al. (2017) data, and that were observed in the single test of autobiographical events as observed by Moreton and Ward.

Our findings confirm that long-term episodic memory is sensitive to serial position and temporal contiguity effects, benchmark findings that are normally considered to be phenomena of immediate free recall. These findings are broadly consistent with unitary memory models that assume that the same episodic memory mechanisms operate at both immediate and long-term memory tasks. Some unitary models assume that to-be-remembered items are associated with a gradually evolving temporal context (TCM; e.g., Howard & Kahana, 2002; Lohnas et al., 2015; Polyn et al., 2009; Sederberg et al., 2008). As the presentation of the study list proceeds, so the temporal context will evolve such that the context associated with neighboring list items will be more similar than the temporal context associated with more distant list items. The evolution of the temporal context is assumed to continue throughout the list, such that at test, the end of list temporal context will be more similar to that associated with the end of list items than earlier list items. These models predict that participants will initially tend to recall recent items first (owing to the greater similarity between the end of list context and the context associated with recency items), but once an item has been recalled, the similarity in contexts between neighboring items will tend to yield temporal contiguity effects. Other unitary memory models claim that multidimensional, to-be-remembered items are encoded along a continuous temporal dimension (e.g., Brown et al., 2007; Neath & Brown, 2006). These models assume that participants’ subjective perception of time is logarithmically compressed, such that more recent events will appear more temporally separated (and so more temporally distinct) than more distant events (which will co-occur in a more temporally crowded region). These models do not, as yet, have a fully implemented account of output order (Brown, Chater, & Neath, 2008).

Although there are similarities between immediate and long-term free recall, it is important to note that there are also some differences. First, the participants in the present experiments showed more of a marked tendency to initiate recall with the first list item, whereas in a test of immediate free recall of eight words, there would be expected to be a stronger tendency to initiate recall with one of the last four list items (e.g., Ward et al., 2010). Although it is true that there is often more primacy and less recency on the first trial of a set of trials (e.g., Unsworth, Brewer, & Spillers, 2011; Wright, 1982), the lack of recency has also been noticed in multitrial long-term free-recall studies in which participants have received up to 50 successive study–test lists (Cortis Mack et al., 2017). The tendency to start with the first list item, coupled with strong temporal contiguity effects, is probably responsible for the stronger primacy than recency effects observed in such aggregate serial position curves. Alternatively, one might wish to argue that an additional contribution from short-term memory is necessary in order to account for the greater recency effect observed in immediate free recall (e.g., Davelaar et al., 2005; Lehman & Malmberg, 2013; Raajimakers, 1993; Unsworth & Engle, 2007).

### Methodological contribution from comparison of smartphone applications

Miller (2012) has cogently argued that psychologists should more frequently make use of smartphones for gathering precise and objective data over sustained periods of time. He has argued that psychologists should leverage the billions of dollars spent each year on smartphone research and development, as it becomes increasingly possible to complete surveys and perform experiments in the wild and in situ that would previously be performed only in the laboratory or when sat by a desktop computer. Psychologists have been slow to build smartphone apps for conducting our experiments. Although it is true that one loses some control over what a participant might be doing when they receive a notification, the remote nature of the smartphone and the increasing ownership (e.g., Smith, 2015) allows researchers new possibilities for presenting and testing stimuli over extended time periods.

In the case of testing long-term free recall, it is self-evident that it is at best inconvenient and at worst impractical for stimuli to be presented to participants in the laboratory in studies such as ours when the ISIs and retention intervals are temporally extended. Other available methods, such as sending stimuli by SMS text messages or emails, have the disadvantage of leaving a permanent copy of the stimuli that could be reviewed by the participant prior to test. By contrast, both the smartphone applications that we have used provide a method for presenting experimentally controlled stimuli over a sustained period of time that disappear shortly after they have been viewed.

There are a large number of methodological advantages for using RECAPP-XPR rather than Snapchat to conduct these experiments. The primary advantages are the ease with which a new RECAPP-XPR study can be set up and the automated nature of the stimulus presentation and recall. A complete spreadsheet of all the stimuli to be presented to all the participants, and a complete data record of all the responses made by all the participants can be extracted from the web-based interface of RECAPP-XPR with single button presses. The secondary advantage is that RECAPP-XPR provides more control and knowledge of when the presented stimuli are actually viewed. Not only does RECAPP-XPR record the precise times at which the words were rated by participants, it also only allows participants to view the stimuli within a specified time period after the presentation of each item.

The primary disadvantage of using RECAPP-XPR is that of its availability. It is currently only available for the iPhone (whereas Snapchat is multiplatform), and it is currently only in beta testing, so it must be published in TestFlight and installed by invitation via iTunes Connect, rather than being universally downloadable from the Apple Store. A secondary disadvantage is that although it makes some choices available for the presentation of stimuli, the RECAPP-XPR application currently does not yield the complete flexibility to readily present every possible schedule of stimuli.

It is worth noting that other software exists, designed specifically for psychological experiments, that could be used with mobile devices. For example, jsPsych is a JavaScript library that can be used to run lab-like behavioral experiments through a web browser. Although jsPsych offers considerable flexibility to the experienced programmer, RECAPP-XPR offers an end-to-end solution that is able to run memory studies on mobile devices for researchers with no programming knowledge.

### The effect of flexibility of stimulus presentations on encoding and recall

One of the advantages of RECAPP-XPR is that it allows for a greater flexibility of stimulus presentation, such that participants are presented with stimuli over much longer periods than is possible in laboratory studies. In Experiment 1, we presented participants with words at a rate of one word every hour, and each stimulus was available for 55 min after notification (e.g., from 10:00 to 10:55 for the first presented word). When participants view and rate the word, they are required to tap the “Finish” tab on the top right-hand side of their smartphone screen and this creates a record for each individual response. Each individual response is time-stamped alongside the response to the orientation question, and is later updated with the recalled words and their respective output order. Once participants finish the trial by terminating their recall, these response records are automatically uploaded to the RECAPP-XPR portal website, as soon as the smartphone is connected to the internet.

Despite its advantages, the fact that participants can view the stimuli within 55 min can present some challenges. One concern is that perhaps participants would view the stimuli for the entire 55-min duration rather than simply reading the word out loud and pressing “Finish.” To examine the validity of this concern, we looked at the timestamp data from Experiment 1. We have a total of 432 response time records (about 76.1% of all stimuli). The majority of the missing data were due to participants not interacting with the individual stimuli or not completing the recall phase, but for technical reasons, RECAPP-XPR failed to record the timestamp on a further 72 stimuli. Of the 432 responses for which we have timestamp data, the majority (271 responses, or 62.7%, as compared to 56.8% in Cortis Mack et al., 2017) were made within 6 min of the stimulus becoming available, and only nine stimuli (2.1%, as compared to 2.6% in Cortis Mack et al., 2017) were viewed in the last 6 min. These data therefore address the first concern and show that participants were not viewing the stimuli for the whole 55 min, but rather, viewed the words as soon as they were available and pressed the “Finish” button soon after.

A second concern is that participants could systematically delay the viewing of alternate words so as to minimize the time between the two stimuli (e.g., by waiting until the last few minutes to submit Word 1 at 10:55, and then submitting Word 2 at 11:00, thus reducing the ISI to 5 min). If participants were deliberately shortening the ISI, this would greatly compromise our interpretation of the temporal contiguity effects observed in Experiment 1. Fortunately, we could again use the timestamp data to verify that the lag +1 responses were not the result of systematically shortened ISIs. We have response time data for 65 of the 70 lag +1 transitions in Experiment 1 (92.9%), and the average time between these successive responses was 60 min 39 s. It is noteworthy that only 13 of these lag +1 transitions were between stimuli viewed less than 45 min apart (and only one pair had been viewed within 15 min of each other). By contrast, the majority of the lag +1 responses (41 out of 65 transitions, or 63.1%, relative to 50.6% in Cortis Mack et al., 2017) were between stimuli responded to at intervals between 50 and 70 min. Consequently, our response time data clearly address the second concern, and similar to Cortis Mack et al. (2017), rule out the possibility that the temporal contiguity effect arises though participants strategically reducing the functional ISI.

### Future projects using RECAPP-XPR

A number of future studies involving RECAPP-XPR are already planned. In one set of experiments, we plan to examine the free recall of word lists presented under incidental learning conditions. It is of interest whether we can obtain the strong temporal contiguity effects observed in the present study when participants have no incentive to try to recall earlier list items during the presentation of later list items. Although temporal contiguity effects are widely observed (e.g., Healey & Kahana, 2014) and have been observed at both short and long interpresentation intervals, there remains the possibility that these effects emerge because participants are reminded to recall earlier items during the presentation of later items (Hintzman, 2011, 2016), such that the temporal contiguity effects are based on associations formed during encoding and are not generated at retrieval. By contrast, temporal context models (Howard & Kahana, 2002; Lohnas et al., 2015; Polyn et al., 2009; Sederberg et al., 2008) assume that items are associated at encoding with temporal context that evolves during the presentation of a list. At test, the associated context is assumed to be reinstated during the successful retrieval of an item, and it is this retrieved context that facilitates retrieval of temporally contiguous items. Our planned studies, in which word lists would be presented under incidental learning conditions, would provide a good test of whether temporal contiguity effects can be generated at retrieval in the absence of associations created during encoding.

We additionally plan to examine the free recall of word lists presented over successive days. This will allow us to examine the free recall of longer lists and will allow us to examine whether our present, primacy-dominated findings are the result of established retrieval strategies for recalling what has happened in one’s day. Arguably, we commonly reflect or are asked about our daily activities toward the end of a day, and we might naturally or be expected to initiate recall with what occurred at the start of the day. By extending the list over successive days, we will be able to circumvent the use of this hypothetical retrieval strategy.

More generally, we hope to work on the development of RECAPP-XPR, to allow for the presentation and testing of paired associates in order to reexamine the time course of forgetting in classic A–B, A–C learning paradigms. By modifying the current version of RECAPP-XPR, we will be able to present and test the recall of competing stimulus–response events that are presented with varying ISIs and varying retention intervals.

### Future developments of RECAPP-XPR

RECAPP-XPR is under active development and is currently in a beta testing phase, with researchers and study participants performing free recall, serial recall, and recognition memory experiments over long timescales. During this phase, we continue to refine and enhance the RECAPP-XPR components following an agile, user-centered design approach, by capturing feedback from researchers and study participants in order to improve the user experience. We note, however, that although testing and development are ongoing, installation and deployment of the iOS mobile application is achieved using Apple’s iTunes Connect Testflight distribution framework, and this is cumbersome for both researchers and participants.

We are currently looking to improve the technology readiness of RECAPP-XPR and to facilitate wider uptake of the system within the memory research community. Ideally we would continue to develop an updated version that (1) consolidates our recent learning as part of the testing phase with a small group of memory researchers, (2) provides critical updates to streamline the deployment of the mobile application through the iTunes App Store, and (3) enables the provision of RECAPP-XPR as a fully managed service that allows researchers to focus on implementing studies without the additional burden of hosting and supporting technical components.

To aid in the development and distribution of RECAPP-XPR, we have provided repositories at https://github.com/Recall-Project that include both all the code for the RECAPP-XPR web service and the RECAPP iOS Client application. However, given the need to have both mobile and server-side components operational to support trials, we believe that the best long-term strategy will be to offer RECAPP-XPR as a fully hosted service. We very much welcome approaches from researchers interested in collaborating on the design of RECAPP-XPR, particularly social science and memory recall researchers who may wish to contribute use-cases or participate in projects that utilize the current system. We are already aware of the following requested requirements: (1) multiplatform download from the Apple app store and Google Play store; (2) increased functionality in the media that can be presented (e.g., images, videos) and an increased range of testing methods that can be requested; and (3) increased flexibility and increased experimenter control of exactly what can be presented when, such that an experimenter could simply upload schedules of items to be presented at specified times and would thus allow for both complete and fixed order randomization.

### Summary and conclusions

We have demonstrated the functionality of our RECAPP-XPR application, which can present and then test recall of lists of stimuli presented over extended temporal schedules. We have showcased its ease of use by comparing RECAPP-XPR to a commercially available application, Snapchat. Although Snapchat is more widely available and more commonly used than our application in social day-to-day interactions, using Snapchat for presentation and testing requires excessive demands on experimenter time, and to obtain a required schedule of presentation requires meticulous organization. By contrast, we have shown that RECAPP-XPR offers greater experimental control over the timing of presentation and testing, and more importantly for the experimenter, an experiment in RECAPP-XPR takes only minutes to set up, after which all data presentation and collection is automated. The similar findings observed from both experiments cross-validate the two methods and confirm the presence of extended primacy effects, limited recency effects, and strong temporal contiguity effects in lists of stimuli presented over very extended temporal schedules.

#### Author note

The authors acknowledge the financial support of the UK Engineering and Physical Sciences Research Council (EPSRC), under EPSRC Reference: EP/N028228/1 (PACTMAN).

## Figures and Tables

**Fig. 7:**
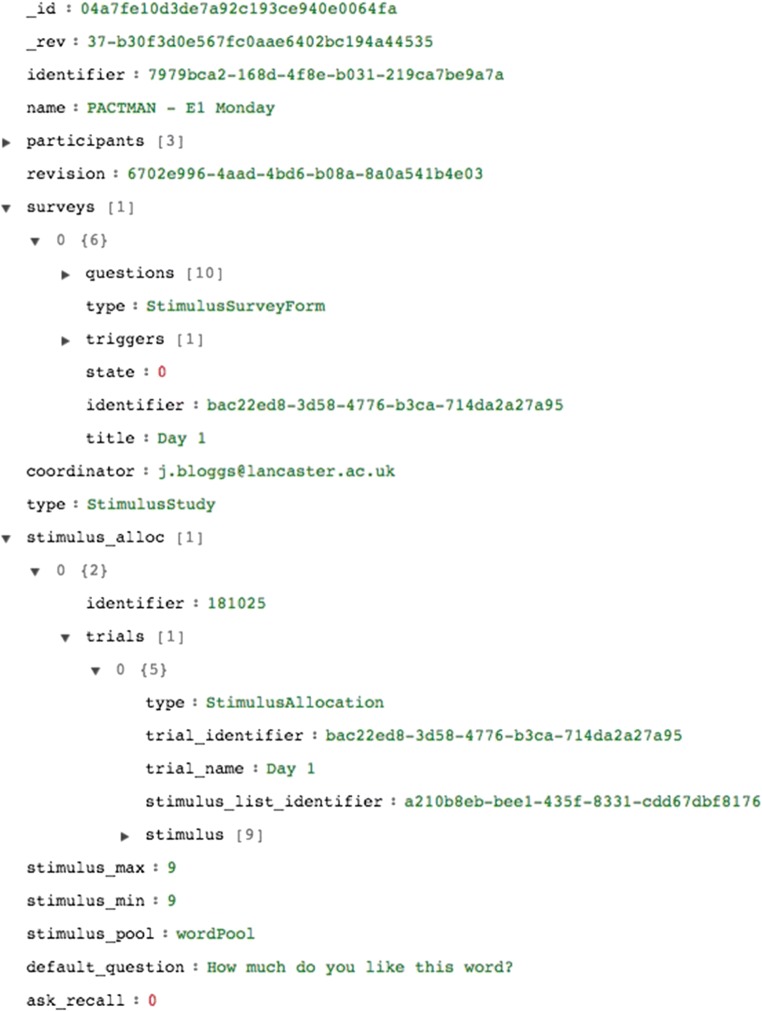
Elements of a JSON schema outlining the project configuration

## Appendix: Technical details about the RECAPP-XPR architecture

### Note 1

The web client’s ProjectModelController processes the experimenter-selected parameters and generates a JSON-formatted schema document (see Fig. 7) describing the configuration of the project and the associated surveys. Once initialization is complete, the module uploads and stores the project schema in the RECAPP-XPR data store, based on CouchDB, a schema-less, document-oriented database (http://guide.couchdb.org). Consistency of the project schema documents is managed and maintained through CouchDB’s revision and conflict model (http://guide.couchdb.org/draft/conflicts.html). The experimental parameters selected via the web management interface rely on the web server returning the latest document revision identifier. In this manner, subsequent edits (e.g., adding new project participants or survey triggers) can be applied through the web interface. When a temporal trigger is selected, the underlying JSON representation of the trigger is attached to the project schema document and updated on the server using the latest revision identifier.

### Note 2

Secure participant and experimenter sessions within the RECAPP-XPR framework are supported and managed through CouchDB’s cookie-based authentication module (http://docs.couchdb.org/en/2.0.0/api/server/authn.html). Specifically, once a participant account registration request is completed via the mobile application, the RECAPP-XPR web service returns a cookie token that allows the application to authenticate future service requests. The authentication cookie is set without an expiration timestamp, facilitating a single “sign-on” mechanism managed by the mobile participant authentication module. This removes the need for participants to log in on subsequent interactions with the application. Furthermore, a long-running session is particularly important for supporting memory experiments in which survey stimulus questions are configured with a short time duration before expiration. After registration, the coordinator can associate the participant with an existing project through the RECAPP-XPR management interface using the participant’s PIN ID. The web client ProjectModelController signals the stimulus allocation module to generate the required stimulus words for the participant and includes the participant’s stimulus allocations and PIN identifier within the project JSON descriptor document locally. The modified descriptor document is then uploaded to the server and stored in the database.

### Note 3

Each available project is returned in a JSON array that is processed by the SurveyProcessor module. This module first identifies each element of the project’s JSON schema document, and the survey, trigger, and stimulus question configurations are identified and stored as native iOS Core Data objects. Second, the module evaluates each survey’s associated temporal triggers to assess whether the survey is expired, completed, active, or available.

## Footnotes

1 The recall app, RECAPP, was itself developed from an early experience-sampling app, XPR—hence the preferred name RECAPP-XPR, which reflects both potential uses.

## References

[CR1] Atkinson RC, Shiffrin RM, Spence KW, Spence JT (1968). Human memory: A proposed system and its control processes. The psychology of learning and motivation: Advances in research and theory.

[CR2] Atkinson RC, Shiffrin RM (1971). The control of short-term memory. Scientific American.

[CR3] Baddeley A, Eysenck MW, Anderson AC (2014). Memory.

[CR4] Baddeley AD (1976). The psychology of memory.

[CR5] Baddeley AD (1986). Working memory.

[CR6] Baddeley AD, Hitch GJ, Dornic S (1977). Recency re-examined. Attention and performance VI.

[CR7] Bjork RA, Whitten WB (1974). Recency-sensitive retrieval processes in long-term free recall. Cognitive Psychology.

[CR8] Brown GDA, Chater N, Neath I (2008). Serial and free recall: Common effects and common mechanisms? A reply to Murdock (2008). Psychological Review.

[CR9] Brown GDA, Neath I, Chater N (2007). A temporal ratio model of memory. Psychological Review.

[CR10] Camos V, Barrouillet P (2014). Working memory: Loss and reconstruction.

[CR11] Conway ARA, Kane MJ, Bunting MF, Hambrick DZ, Wilhelm O, Engle RW (2005). Working memory span tasks: A methodological review and user’s guide. Psychonomic Bulletin & Review.

[CR12] Cortis Mack C, Cinel C, Davies N, Harding M, Ward G (2017). Serial position, output order, and list length effects for words presented on smartphones over very long intervals. Journal of Memory and Language.

[CR13] Crowder RG (1976). Principles of learning and memory.

[CR14] da Costa Pinto AAN, Baddeley AD (1991). Where did you park your car? Analysis of a naturalistic long-term recency effect. European Journal of Cognitive Psychology.

[CR15] Davelaar EJ, Goshen-Gottstein Y, Ashkenazi A, Haarmann HJ, Usher M (2005). The demise of short-term memory revisited: Empirical and computational investigations of recency effects. Psychological Review.

[CR16] Deese J (1957). Serial organisation in the recall of disconnected items. Psychological Reports.

[CR17] Deese J, Kaufman RA (1957). Serial effects in recall of unorganized and sequentially organized verbal material. Journal of Experimental Psychology.

[CR18] Friendly M, Franklin PE, Hoffman D, Rubin DC (1982). The Toronto Word Pool: Norms for imagery, concreteness, orthographic variables, and grammatical usage for 1,080 words. Behavior Research Methods & Instrumentation.

[CR19] Glanzer M, Bower GH (1972). Storage mechanisms in recall. The psychology of learning and motivation: Advances in research and theory.

[CR20] Glanzer M, Cunitz AR (1966). Two storage mechanisms in free recall. Journal of Verbal Learning and Verbal Behavior.

[CR21] Greene RL (1992). Human memory: Paradigms and paradoxes.

[CR22] Healey MK, Kahana MJ (2014). Is memory search governed by universal principles or idiosyncratic strategies?. Journal of Experimental Psychology: General.

[CR23] Hintzman DL (2011). Research strategy in the study of memory: Fads, fallacies, and the search for the coordinates of truth. Perspectives on Psychological Science.

[CR24] Hintzman DL (2016). Is memory organized by temporal contiguity?. Memory & Cognition.

[CR25] Hitch GJ, Ferguson J (1991). Prospective memory for future intentions: Some comparisons with memory for past events. European Journal of Cognitive Psychology.

[CR26] Hogan RM (1975). Interitem encoding and directed search in free recall. Memory & Cognition.

[CR27] Howard MW, Kahana MJ (1999). Contextual variability and serial position effects in free recall. Journal of Experimental Psychology: Learning, Memory, and Cognition.

[CR28] Howard MW, Kahana MJ (2002). A distributed representation of temporal context. Journal of Mathematical Psychology.

[CR29] Jahnke JC (1965). Primacy and recency effects in serial-position curves of immediate recall. Journal of Experimental Psychology.

[CR30] Kahana MJ (1996). Associative retrieval processes in free recall. Memory & Cognition.

[CR31] Kahana MJ (2012). Foundations of human memory.

[CR32] Laming D (1999). Testing the idea of distinct storage mechanisms in memory. International Journal of Psychology.

[CR33] Lehman M, Malmberg KJ (2013). A buffer model of memory encoding and temporal correlations in retrieval. Psychological Review.

[CR34] Lohnas LJ, Polyn SM, Kahana MJ (2015). Expanding the scope of memory search: Modeling intralist and interlist effects in free recall. Psychological Review.

[CR35] Maylor EA, Chater N, Brown GDA (2001). Scale invariance in the retrieval of retrospective and prospective memories. Psychonomic Bulletin & Review.

[CR36] Miller G (2012). The smartphone psychology manifesto. Perspectives on Psychological Science.

[CR37] Moreton BJ, Ward G (2010). Time scale similarity and long-term memory for autobiographical events. Psychonomic Bulletin & Review.

[CR38] Murdock BB (1962). The serial position effect of free recall. Journal of Experimental Psychology.

[CR39] Murdock BB (1974). Human memory: Theory and data.

[CR40] Neath I, Brown GDA, Ross BH (2006). SIMPLE: Further applications of a local distinctiveness model of memory. The psychology of learning and motivation (Vol. 46).

[CR41] Neath I, Surprenant AM (2003). Human memory.

[CR42] Piwek L, Joinson A (2016). What do they snapchat about? Patterns of use in time-limited instant messaging service. Computers in Human Behavior.

[CR43] Polyn SM, Norman KA, Kahana MJ (2009). A context maintenance and retrieval model of organizational processes in free recall. Psychological Review.

[CR44] Postman L, Phillips LW (1965). Short-term temporal changes in free recall. Quarterly Journal of Experimental Psychology.

[CR45] Raaijmakers JGW, Meyer DE, Kornblum S (1993). The story of the two-store model of memory: Past criticisms, current status, and future directions. Attention and performance XIV: Synergies in experimental psychology, artificial intelligence, and cognitive neuroscience.

[CR46] Raaijmakers JGW, Shiffrin RM (1981). Search of associative memory. Psychological Review.

[CR47] Rubin DC (1982). On the retention function for autobiographical memory. Journal of Verbal Learning and Verbal Behavior.

[CR48] Rundus D (1971). Analysis of rehearsal processes in free recall. Journal of Experimental Psychology.

[CR49] Sederberg PB, Howard MW, Kahana MJ (2008). A context-based theory of recency and contiguity in free recall. Psychological Review.

[CR50] Smith A (2015). The smartphone difference (Report of the Pew Internet & American Life Project).

[CR51] Statista (2018). Number of daily active Snapchat users from 1st quarter 2014 to 4th quarter 2017 (in millions). Retrieved from https://www.statista.com/statistics/545967/snapchat-app-dau/

[CR52] Sumby WH (1963). Word frequency and serial position effects. Journal of Verbal Learning and Verbal Behavior.

[CR53] Tzeng OJL (1973). Positive recency effects in delayed free recall. Journal of Verbal Learning and Verbal Behavior.

[CR54] Unsworth N, Brewer GA, Spillers GJ (2011). Inter-and intra-individual variation in immediate free recall: An examination of serial position functions and recall initiation strategies. Memory.

[CR55] Unsworth N, Engle RW (2007). The nature of individual differences in working memory capacity: Active maintenance in primary memory and controlled search from secondary memory. Psychological Review.

[CR56] Unsworth N, Redick TS, Heitz RP, Broadway JM, Engle RW (2009). Complex working memory span tasks and higher-order cognition: A latent-variable analysis of the relationship between processing and storage. Memory.

[CR57] Usher M, Davelaar EJ, Haarmann HJ, Goshen-Gottstein Y (2008). Short-term memory after all: Comment on Sederberg, Howard, and Kahana (2008). Psychological Review.

[CR58] Ward G, Tan L, Grenfell-Essam R (2010). Examining the relationship between free recall and immediate serial recall: The effects of list length and output order. Journal of Experimental Psychology: Learning, Memory, and Cognition.

[CR59] Wright RE (1982). Adult age similarities in free recall output order and strategies. Journal of Gerontology.

